# Clinical outcomes of patients with EGFR-mutated NSCLC developing interstitial lung disease during first-line osimertinib therapy: a sub-analysis of the Reiwa study

**DOI:** 10.1093/jjco/hyae178

**Published:** 2024-12-20

**Authors:** Takayuki Kobayashi, Kageaki Watanabe, Yukio Hosomi, Kiyotaka Yoh, Kazuhiro Usui, Kazuma Kishi, Go Naka, Shu Tamano, Kohei Uemura, Hideo Kunitoh

**Affiliations:** Department of Thoracic Oncology and Respiratory Medicine, Tokyo Metropolitan Cancer and Infectious Diseases Center, Komagome Hospital, Tokyo, Japan; Department of Thoracic Oncology and Respiratory Medicine, Tokyo Metropolitan Cancer and Infectious Diseases Center, Komagome Hospital, Tokyo, Japan; Department of Thoracic Oncology and Respiratory Medicine, Tokyo Metropolitan Cancer and Infectious Diseases Center, Komagome Hospital, Tokyo, Japan; Department of Thoracic Oncology, National Cancer Center Hospital East, Chiba, Japan; Respiratory Medicine, NTT Medical Center Tokyo, Tokyo, Japan; Department of Respiratory Medicine, Toho University Omori Medical Center, Tokyo, Japan; Department of Respiratory Medicine, National Center for Global Health and Medicine, Tokyo, Japan; Department of Biostatistics and Bioinformatics Course, Graduate School of Interdisciplinary Information Studies, The University of Tokyo, Tokyo, Japan; Department of Biostatistics and Bioinformatics, The Interfaculty Initiative in Information Studies, The University of Tokyo, Tokyo, Japan; Department of Chemotherapy, Japan Red Cross Medical Center, Tokyo, Japan

**Keywords:** osimertinib, EGFR, non-small cell lung cancer, interstitial lung disease, post-osimertinib therapy

## Abstract

**Introduction:**

Osimertinib-induced interstitial lung disease in untreated *EGFR*-mutated, advanced non-small cell lung cancer is being reported at a higher rate in Japan than elsewhere. However, data on the interstitial lung disease incidence during first-line osimertinib therapy and the course of lung cancer treatments administered after interstitial lung disease onset in the real-world setting are scarce.

**Materials and Methods:**

The present study reviewed the data from the Reiwa study, a multicentric, observational study examining the efficacy and safety of first-line osimertinib therapy in the clinical setting. Patients with *EGFR*-mutated non-small cell lung cancer who began osimertinib therapy between September 2018 and August 2020 were enrolled and followed until August 2022.

**Results:**

Among 583 patients receiving first-line osimertinib therapy, 75 (12.8%) had interstitial lung disease development, and 18 (3.0%) had at least grade 3 interstitial lung disease. Fifty-nine patients (78%) received some form of treatment following interstitial lung disease onset. An epidermal growth factor receptor-tyrosine kinase inhibitor rechallenge was performed in 31 patients (41%), with 18 (24%) receiving osimertinib again. Interstitial lung disease recurred in five (28%) of these 18 patients, none of 13 patients receiving another type of tyrosine kinase inhibitor, and seven (25%) of 28 patients receiving chemotherapy and/or immune checkpoint inhibitor therapy. The median overall survival after the initial osimertinib therapy was 38.4 months and 12.2 months for patients with interstitial lung disease grade 1–2 and grade 3–4, respectively (hazard ratio: 0.37; 95% confidence interval: 0.20–0.70; *P* = 0.002).

**Conclusion:**

Patients with interstitial lung disease grade 3–4 had poorer survival during the first-line osimertinib therapy. A substantial risk of interstitial lung disease recurrence was associated with post-osimertinib therapy.

**Trial registration code**: UMIN000038683.

## Teaser

Grade 3–4 osimertinib-induced interstitial lung disease in epidermal growth factor receptor-mutated non-small cell lung cancer significantly reduced survival compared to lower grades, with substantial interstitial lung disease recurrence risk upon subsequent therapy.

## Introduction

Osimertinib, a third-generation epidermal growth factor receptor tyrosine kinase inhibitor (EGFR-TKI), has become the standard, first-line treatment for advanced non-small cell lung cancer (NSCLC) with epidermal growth factor receptor (EGFR) mutations. This shift in the treatment paradigm is based on the superior efficacy of the current types of EGFR-TKI compared to the earlier forms, as demonstrated in the FLAURA trial [11, 14] by significantly longer progression-free survival (PFS) and overall survival (OS). However, osimertinib is associated with a significant increase in the incidence of interstitial lung disease (ILD), particularly among Japanese patients. In the international cohort of the FLAURA trial, the ILD incidence was 4% compared to the 12.3% observed in a Japanese subpopulation [10]. The ILD incidence was also 12.8% in the OSI-FACT study [12], a retrospective, observational study enrolling Japanese patients. This higher ILD incidence in Japanese patients compared to other populations underscores the importance of careful monitoring and clinical management of this potentially serious adverse event.

Extensive research has been conducted on the association of EGFR-TKIs with lung toxicity. However, while previous studies have examined osimertinib-related ILD in second or later lines of treatment in Japanese patients [2], studies focusing solely on osimertinib-induced ILD in the first-line setting are scarce. Past studies were more concerned with radiological assessment, patient characteristics, and risk factors of ILD development, including transient asymptomatic pulmonary opacities (TAPO) [5, 9, 13]. However, there remains a significant gap in our understanding of the prognosis of patients in relation to ILD severity, as well as in the patterns, efficacy, and safety of treatments following ILD onset. Moreover, the clinical implications of TAPO during first-line osimertinib therapy, including its impact on treatment continuation, are not fully known [7]. These knowledge gaps underscore the need for a comprehensive, real-world study examining the clinical outcomes of patients with ILD development during first-line osimertinib therapy, including a detailed analysis of later treatments and their outcomes. The present study therefore aimed to address these critical gaps in our understanding of first-line osimertinib-induced ILD in EGFR-mutated NSCLC though investigating the ILD incidence, efficacy and safety of treatments administered after osimertinib discontinuation, prognosis of patients with ILD development during osimertinib therapy, and resumption of osimertinib therapy after ILD onset.

## Material and methods

### Study design and patients

The present, multicentric, prospective, and observational study used the data collection methods outlined in the Reiwa Study protocol [15]. Patients aged 20 years or older with a diagnosis of advanced or recurrent EGFR mutation-positive NSCLC who were scheduled to receive EGFR-TKI therapy were enrolled. This study was conducted in 30 Japanese centers from September 2018 to August 2020, with follow-up continuing until August 2022.

Patients with ILD development following their initial osimertinib monotherapy were included. These patients were followed up periodically every 6 months using case report forms to assess the clinical efficacy and safety of their ongoing treatment as well as post-osimertinib therapy.

The present study complies with the principles of the Helsinki Declaration and the Japanese ethics guidelines [1] for medical and biological research involving human subjects. The Ethics Review Committee of the Japanese Red Cross Medical Center (26 April 2019, order number 976) and the relevant committee at each participating center approved this study. All the patients provided their written informed consent prior to enrollment.

### Definitions and assessments

The disease stages were classified in accordance with the Union for International Cancer Control TNM classification system, 8^th^ edition. The National Cancer Institute Common Terminology Criteria for Adverse Events, version 5.0 was used to ascertain the ILD diagnosis and onset data. The investigators performed chest imaging based on the patient’s clinical condition. The diagnosis of ILD was made by the investigators.

PFS was defined as the period from the start of osimertinib therapy to the onset of progressive disease (PD). OS was defined as the period from the start of osimertinib therapy to death from any cause. Progression was determined by the investigators using the Response Evaluation Criteria in Solid Tumors, version 1.1. Patients lost to follow-up during the observation period were classified as censored on the date of discontinuation. Those who did not show progression during the observation period were classified as censored on the date of the final confirmation. For post-osimertinib treatments, OS was calculated from the date at which the treatment was begun. An EGFR-TKI rechallenge was defined as the re-administration of any EGFR-TKI, including osimertinib, as a second-line treatment following the initial onset of pneumonitis induced by the first-line osimertinib therapy.

### Outcome measures

The present study focused on the following outcome measures: patient background characteristics, the ILD incidence in patients receiving first-line osimertinib therapy, ILD severity grade distribution, treatments after ILD onset, survival analysis in patients with ILD stratified by severity grade, and the efficacy and safety of subsequent treatments. No data on ILD treatments, including corticosteroid use, were available.

### Statistical analysis

All statistical analyses were based on an ILD analysis set and included the 95% confidence interval (CI). Survival analysis used the Kaplan–Meier method and hazard ratio (HR), and the 95% CI was estimated using the Cox proportional hazards model. The chi-square test or Fisher’s exact test was used to analyze categorical variables as appropriate. Descriptive statistics were used to summarize the patient and tumor characteristics. Two-sided *P* < 0.05 was considered to indicate statistical significance. All the statistical analyses were performed using R version 4.4.1 (R Core Team, Vienna, Austria).

## Results

### Patient background characteristics

Of the 583 patients who received osimertinib monotherapy as their first-line treatment, 75 patients (12.8%) experienced ILD development. Table 1 summarizes their baseline characteristics. Their median age was 75 years (range: 50–88 years), and female patients comprised the majority of the cohort (60% vs. 40%). Most patients had no history of smoking (53%) or were former smokers (43%), and only 4% were current smokers. The baseline characteristics of the patients with ILD development and those of the entire cohort did not differ significantly.

**Table 1:** Baseline clinical characteristics of the patients

**Characteristics**	**Total**	**With ILD**	***P* value**
	**(N = 583)**	**(N = 75)**	
Age, years			0.004
Median (Range)	72 (30–95)	75 (50–88)	
Sex, n (%)			0.89
Male	224 (38.4)	30 (40)	
Female	359 (61.6)	45 (60)	
Smoking status, n (%)			0.68
Never	325 (55.8)	40 (53)	
Current	34 (5.8)	3 (4)	
Former	224(38.4)	32 (43)	
ECOG PS, n (%)			0.86
0	216 (37.1)	30 (40)	
1	281 (48.2)	37 (49.4)	
2	60 (10.3)	7 (9.3)	
3	20 (3.4)	1 (1.3)	
4	2 (0.3)	0 (0)	
Missing	4 (0.7)	0 (0)	
Histological type, n (%)			1.00
Adenocarcinoma	571 (98)	74 (98.7)	
Squamous	9 (1.5)	1 (1.3)	
Others	3 (0.5)	0 (0)	
Clinical Stage, n (%)			0.84
IV	393 (67.4)	52 (69.3)	
Recurrence	190 (32.6)	23 (30.7)	
EGFR mutation type, n (%)			0.44
Exon 19 deletion	285 (48.9)	32 (43)	
L858R	266 (45.6)	40 (53)	
Others	32 (5.5)	3 (4.0)	
Comorbidity, n (%)			
ILD	3 (0.5)	1 (1.3)	0.95
Emphysema	23 (4.0)	3 (4.0)	1.00
Hepatic disease	22 (3.8)	3 (4.0)	1.00
Previous treatment, n (%)			
Chemotherapy	85 (14.6)	7 (9.3)	0.29
Adjuvant/Neoadjuvant chemotherapy	27 (4.65)	5 (6.7)	
Chemoradiotherapy	58 (9.95)	2 (2.7)	
Lung operation	187 (32.1)	20 (27)	0.41
Radiotherapy	114 (19.6)	16 (21.3)	0.83
Intrathoracic	63 (10.8)	10 (13.3)	
Extrathoracic	51 (8.8)	6 (8.0)	

Abbreviations: ECOG, Eastern Cooperative Oncology Group; PS, performance status; EGFR, epidermal growth factor receptor; ILD, interstitial lung disease.

### Incidence and distribution of ILD severity grade

The severity grade distribution was as follows: 21 (28.0%), 36 (48.0%), 12 (16.0%), and six (8.0%) patients had Grade 1, Grade 2, Grade 3, and Grade 4 severity, respectively. No patient had Grade 5 ILD. Overall, 18 of the 75 patients (24%) had Grade 3 or worse ILD, which corresponded to 3.0% of the total cohort receiving first-line osimertinib therapy. The median time from the initial osimertinib administration to ILD onset was 2.5 months (range: 0.2–25.4 months). Table 2 presents the median time to ILD onset stratified by the ILD severity grade.

**Table 2:** ILD grade and time to disease onset

**ILD Grade**	**Number (%)**	**Median time in months (range)**
Grade 1	21 (28.0)	3.0 (0.4–25.2)
Grade 2	36 (48.0)	2.1 (0.2–25.4)
Grade 3	12 (16.0)	1.4 (0.3–23.5)
Grade 4	6 (8.0)	4.5 (0.3–25.4)
All grades	75 (100)	2.5 (0.2–25.4)

Abbreviations: ILD, interstitial lung disease.

### Treatments after ILD onset

After osimertinib was discontinued due to ILD onset, 59 of the 75 patients (78%) received further treatment. The post-osimertinib treatments comprised the following: an EGFR-TKI rechallenge in 31 patients (41% of all patients with ILD), with 18 patients (24%) receiving an osimertinib rechallenge and 13 patients (17%) receiving some other EGFR-TKI; of the 28 patients (37%) who received a non-EGFR-TKI treatment, 17 patients (22.4%) received cytotoxic chemotherapy, nine patients (12%) received cytotoxic chemotherapy + vascular endothelial growth factor inhibitor (VEGFI), one patient (1.3%) received an immune checkpoint inhibitor (ICI), and one patient (1.3%) received ICI + cytotoxic chemotherapy + VEGFI (Supplementary Table S1). Table 3 details the types of post-osimertinib treatment administered per initial ILD grade. Supplementary Fig. S1 illustrates the sequence of events in 59 patients who received subsequent treatment following ILD development during osimertinib treatment, stratified by ILD severity grades. The median time from ILD onset to the start of the subsequent treatment was 2.3 months (range: 0–26.6) (Supplementary Fig. S1). Of the 59 patients who received post-osimertinib treatment, 24 (41%) experienced disease progression by the time the treatment was begun. EGFR-TKI rechallenge was primarily initiated after ILD improvement but before disease progression confirmation in most cases, although some patients started rechallenge after both ILD improvement and disease progression were confirmed. The specific timing of rechallenge was determined by the investigators based on individual patient factors.

**Table 3:** Second-line treatments by initial ILD grade in first-line osimertinib-induced ILD

**Initial ILD Grade**	**Type of second-line treatment**						
	Osimertinib	EGFR-TKI other than osimertinib	Cytotoxic	Cytotoxic + VEGFI	ICI	ICI + cytotoxic + VEGFI	No treatment
Grade 1 (N = 21)	10	4	2	1	1	0	3
Grade 2 (N = 36)	6	6	12	5	0	1	6
Grade 3 (N = 12)	2	3	2	3	0	0	2
Grade 4 (N = 6)	0	0	1	0	0	0	5
All grades (N = 75)	18	13	17	9	1	1	16

Abbreviations: EGFR, epidermal growth factor receptor; TKI, tyrosine kinase inhibitor; ILD, interstitial lung disease; VEGFI, vascular endothelial growth factor inhibitor; ICI, immune checkpoint inhibitor.

The ILD recurrence rate among patients receiving the EGFR-TKI rechallenge was 16% (5/31 patients). Notably, all the recurrences were in patients receiving the osimertinib rechallenge, who had a recurrence rate of 28% (5/18 patients). No recurrence was observed in any of the patients receiving other EGFR-TKIs (0/13 patients). In comparison, patients receiving chemotherapy and/or immunotherapy had an ILD recurrence rate of 25% (7/28 patients). Furthermore, among these patients, those receiving a treatment regimen incorporating cytotoxic chemotherapy had a recurrence rate of 23% (6/26 patients) while those receiving a treatment regimen incorporating an ICI had a 50% recurrence rate (1/2 patients). The median time from the start of post-osimertinib treatment to an ILD relapse was 2.3 months (range: 0.17–18.1 months) for all 12 patients with an ILD recurrence, 4.5 months (range: 0.17–18.1 months) for the patients receiving the osimertinib rechallenge, and 2.1 months (range: 0.23–5.07 months) for the patients receiving chemotherapy and/or immunotherapy. Supplementary Table S2 shows the characteristics and the time from the start of post-osimertinib treatment to an ILD relapse in these 12 patients. Among the 12 patients who experienced ILD recurrence, the initial ILD grade were Grade 1 (n = 3), Grade 2 (n = 7), and Grade 3 (n = 2), indicating no clear association between initial ILD grade and recurrence risk. The present study also examined the cumulative incidence of ILD recurrences in patients receiving post-osimertinib treatment following ILD onset. Among the 59 patients, those receiving the osimertinib rechallenge did not differ significantly from the non-EGFR TKI treatment group in terms of the risk of ILD recurrence (hazard ratio [HR]: 1.08; 95% CI: 0.34 to 3.4; *P* = 0.9) (Supplementary Fig. S2).

### Survival analysis in patients with ILD stratified by severity grade

**Figure 1:**
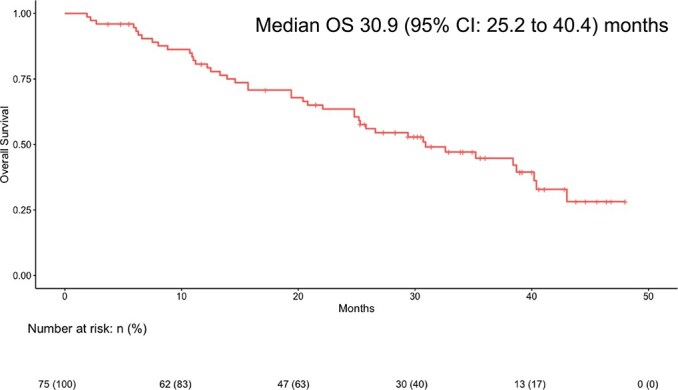
Kaplan–Meier estimates of overall survival (OS) in all the patients with interstitial lung disease during osimertinib therapy (n = 75). OS was calculated starting from the date of osimertinib treatment initiation.

Survival analysis was conducted for all the patients with ILD development during osimertinib therapy. The median OS of the entire ILD cohort was 30.9 months (95% CI: 25.2 to 40.4 months) (Fig. 1).

**Figure 2:**
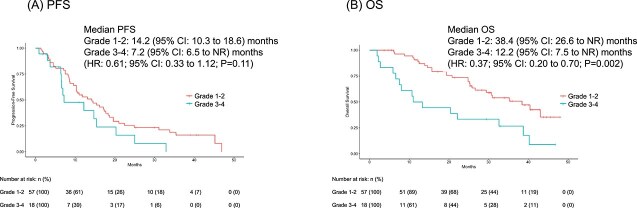
Kaplan–Meier survival estimates of (A) progression-free survival (PFS) and (B) overall survival (OS) in patients with grade 1–2 interstitial lung disease (ILD) (n = 57) vs. those with grade 3–4 ILD (n = 18). PFS and OS were calculated starting from the date of osimertinib treatment initiation.

When stratified by the ILD severity grade, the survival outcomes differed between the patients with Grade 1–2 ILD and those with Grade 3–4 ILD although the patient characteristics of these groups did not change (Table 4). The median PFS was 14.2 months (95% CI: 10.3 to 18.6 months) in the Grade 1–2 group and 7.2 months (95% CI: 6.5 to NR months) in the Grade 3–4 group (HR: 0.61; 95% CI: 0.33 to 1.12; *P* = 0.11) (Fig. 2A). The most pronounced difference was observed in OS, with the Grade 1–2 group achieving a median of 38.4 months (95% CI: 26.6 to NR months) compared to 12.2 months (95% CI: 7.5 to NR months) in the Grade 3–4 group (HR: 0.37; 95% CI: 0.20 to 0.70; *P* = 0.002) (Fig. 2B).

**Table 4:** Patient characteristics in ILD grade 1–2 and grade 3–4 groups.

**Characteristics**		**Grade 1–2**	**Grade 3–4**	***P***
		**(N = 57)**	**(N = 18)**	
Age, n (%)	<75	28 (49.1)	8 (44.4)	0.94
	≧75	29 (50.9)	10 (55.6)	
Sex, n (%)	Female	34 (59.6)	11 (61.1)	1
	Male	23 (40.4)	7 (38.9)	
EGFR type, n (%)	Exon 19 deletion	25 (43.9)	7 (38.9)	0.523
	L858R	29 (50.9)	11 (61.1)	
	Others	3 (5.3)	0 (0.0)	
ECOG PS, n (%)	0–1	53 (93.0)	14 (77.8)	0.166
	≧2	4 (7.0)	4 (22.2)	
Smoking status, n (%)	Ever	26 (45.6)	9 (50.0)	0.957
	Never	31 (54.4)	9 (50.0)	
Comorbidity, n (%)	No	51 (89.5)	17 (94.4)	0.867
	Hepatic disease	3 (5.3)	0 (0.0)	0.761
	Emphysema	2 (3.5)	1 (5.6)	1
	ILD	1 (1.8)	0 (0.0)	1
Clinical stage, n (%)	IV	37 (64.9)	15 (83.3)	
	Recurrent	20 (35.1)	3 (16.7)	0.236
Histological type, n (%)	Adenocarcinoma	56 (98.2)	18 (100)	1
	Squamous	1 (1.8)	0 (0.0)	
Chemotherapy, n (%)	Non	51 (89.5)	17 (94.4)	0.7
	Adjuvant	4 (7.0)	1 (5.6)	
	Chemoradiotherapy	2 (3.5)	0 (0.0)	
Lung operation, n (%)	Yes	16 (28.1)	4 (22.2)	0.854
	No	41 (71.9)	14 (77.8)	
Radiotherapy, n (%)	Non	45 (78.9)	14 (77.8)	0.828
	Intrathoracic	7 (12.3)	3 (16.7)	
	Extrathoracic	5 (8.8)	1 (5.6)	
Post-osimertinib treatment, n (%)	Yes	48 (84.2)	11 (61.1)	0.079
	No	9 (15.8)	7 (38.9)	
Total post-osimertinib treatments, n (%)	0	9 (15.8)	7 (38.9)	0.109
	1–2	37 (64.9)	8 (44.4)	
	≥3	11 (19.3)	3 (16.7)	
Type of second-line treatment, n (%)	No	9 (15.8)	7 (38.9)	0.315
	Osimertinib	16 (28.1)	2 (11.1)	
	EGFR TKI	10 (17.5)	3 (16.7)	
	Cytotoxic	14 (24.6)	3 (16.7)	
	Cytotoxic + VEGFI	6 (10.5)	3 (16.7)	
	ICI	1 (1.8)	0 (0.0)	
	ICI + cytotoxic + VEGFI	1 (1.8)	0 (0.0)	

Abbreviations: EGFR, epidermal growth factor receptor; ECOG, Eastern Cooperative Oncology Group; PS, performance status; TKI, tyrosine kinase inhibitor; ILD, interstitial lung disease; VEGFI, vascular endothelial growth factor inhibitor; ICI, immune checkpoint inhibitor.

### Efficacy of subsequent treatments

The efficacy of the rechallenge with osimertinib and other EGFR-TKIs and the non-EGFR-TKI treatments administered after the discontinuation osimertinib therapy due to ILD onset was analysed. The patient characteristics were generally similar across the treatment groups, with the EGFR-TKI rechallenge group having a higher proportion of elderly patients than the non-EGFR-TKI group (Table 5).

**Table 5:** Patient characteristics in the EGFR TKI rechallenge and non-EGFR TKI rechallenge groups

**Characteristics**		**EGFR TKI rechallenge**	**Non-EGFR-TKI rechallenge**	***P***
		**(N = 31)**	**(N = 28)**	
Age, n (%)	<75	11 (35.5)	19 (67.9)	0.026
	≥75	20 (64.5)	9 (32.1)	
Sex, n (%)	Male	15 (48.4)	10 (35.7)	0.472
	Female	16 (51.6)	18 (64.3)	
EGFR type, n (%)	Exon 19 deletion	13 (41.9)	12 (42.9)	0.774
	L858R	17 (54.8)	14 (50.0)	
	Others	1 (3.2)	2 (7.1)	
ECOG PS, n (%)	0–1	29 (93.5)	27 (96.4)	1
	≥2	2 (6.5)	1 (3.6)	
Smoking status, n (%)	Ever	14 (45.2)	14 (50.0)	0.912
	Never	17 (54.8)	14 (50.0)	
Comorbidity, n (%)	No	28 (90.3)	25 (89.3)	1
	Hepatic disease	0 (0.0)	2 (7.1)	0.427
	Emphysema	2 (6.5)	1 (3.6)	1
	ILD	1 (3.2)	0 (0.0)	1
Clinical stage, n (%)	IV	20 (64.5)	20 (71.4)	0.773
	Recurrence	11 (35.5)	8 (28.6)	
Histologic type, n (%)	Adenocarcinoma	31 (100)	27 (96.4)	0.959
	Squamous	0 (0.0)	1 (3.6)	
Chemotherapy, n (%)	No	27 (87.1)	25 (89.3)	0.94
	Adjuvant	3 (9.7)	2 (7.1)	
	Chemoradiotherapy	1 (3.2)	1 (3.6)	
Lung operation, n (%)	Yes	9 (29.0)	8 (28.6)	1
	No	22 (71)	20 (71.4)	
Radiotherapy, n (%)	Non	21 (67.7)	25 (89.3)	0.125
	Intrathoracic	5 (16.1)	2 (7.1)	
	Extrathoracic	5 (16.1)	1 (3.6)	
ILD grade, n (%)	1–2	26 (83.9)	22 (78.6)	0.851
	3–4	5 (16.1)	6 (21.4)	
Total post-osimertinib treatments, n (%)	1–2	27 (87.1)	18 (64.3)	0.08
	≥3	4 (12.9)	10 (35.7)	
Type of second-line treatment, n (%)	EGFR-TKI	31 (100.0)	0 (0.0)	<0.01
	Cytotoxic	0 (0.0)	17 (60.7)	
	Cytotoxic + VEGFI	0 (0.0)	9 (32.1)	
	ICI	0 (0.0)	1 (3.6)	
	ICI + cytotoxic + VEGFI	0 (0.0)	1 (3.6)	

Abbreviations: EGFR, epidermal growth factor receptor; ECOG, Eastern Cooperative Oncology Group; PS, performance status; TKI, tyrosine kinase inhibitor; ILD, interstitial lung disease; VEGFI, vascular endothelial growth factor inhibitor; ICI, immune checkpoint inhibitor.

**Figure 3:**
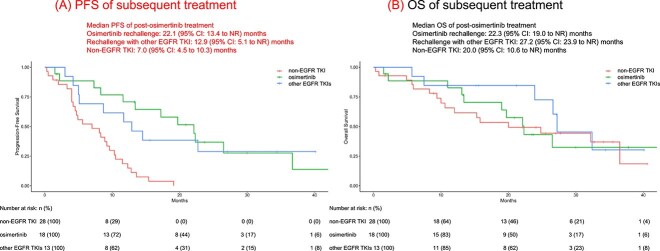
Kaplan–Meier estimates of (A) progression-free survival (PFS) and (B) overall survival (OS) in patients with post-osimertinib treatment. Patients receiving the osimertinib rechallenge (n = 18), rechallenge with other epidermal growth factor receptor tyrosine kinase inhibitor (EGFR TKIs) (n = 13), and non-EGFR TKI rechallenge (n = 28) are compared. PFS and OS in the entire cohort were calculated starting from the date of treatment initiation.

The response evaluation, overall response rate (ORR), and disease control rate (DCR) for each second-line treatment are presented in Supplementary Table S3. The ORR and DCR for the osimertinib rechallenge were 27.8% and 50%, respectively. The median PFS was 22.1 months (95% CI: 13.4 to NR months), 12.9 months (95% CI: 5.1 to NR months), and 7.0 months (95% CI: 4.5 to 10.3 months) in the osimertinib rechallenge, other EGFR-TKI rechallenge, and chemotherapy and/or immunotherapy group, respectively (Fig. 3A). The median OS was 22.3 months (95% CI: 19.0 to NR months), 27.2 months (95% CI: 23.9 to NR months), and 20.0 months (95% CI: 10.6 to NR months) in the osimertinib rechallenge, other EGFR-TKI rechallenge, and chemotherapy and/or immunotherapy group, respectively (Fig. 3B).

## Discussion

The present, prospective study provided some significant findings regarding ILD in patients receiving first-line osimertinib for EGFR-mutated NSCLC. First, the incidence of ILD was 12.8%, with 3.0% of the cases having severity grade 3 or worse, in line with the findings of previous studies of Japanese patients. Second, ILD occurred relatively soon (median: 2.5 months) after the start of the treatment but developed more than a year later in some cases. Third, patients with low severity grade ILD (Grade 1–2) had a better prognosis than those with higher severity grade ILD (Grade 3–4). Finally, the ILD recurrence rate associated with the EGFR-TKI rechallenge was 16% overall, with a higher rate of 28% being associated with the osimertinib rechallenge. ILD was also found to occur during post-osimertinib chemotherapy.

The present, prospective study revealed an incidence of ILD in patients receiving first-line osimertinib therapy for EGFR-mutated NSCLC comparable to previous studies of Japanese patients. Ohe et al. reported an ILD incidence of 12.3% in the Japanese subset of the FLAURA trial [10] while the OSI-FACT study [12] reported a similar incidence of 12.8%, and Kodama et al. [4] reported an ILD incidence of 13% (28 out of 215 patients). The results of the present study further corroborated these findings, suggesting a consistent ILD risk profile for osimertinib in Japanese NSCLC patients. Importantly, the present study found that patients with lower-grade ILD (Grade 1–2) had better a prognosis than those with higher-grade ILD (Grade 3–4). The poorer survival outcomes observed in patients with Grade 3–4 ILD may be partially explained by their limited ability to receive subsequent treatments. As shown in Table 4, only 61.1% of patients with Grade 3–4 ILD received post-osimertinib treatment, compared to 84.2% of those with Grade 1–2 ILD. This treatment limitation likely contributed to the shorter PFS and OS in the Grade 3–4 group. This observation adds valuable information to our current knowledge, as it highlights the prognostic value of ILD severity in patients receiving osimertinib.

The second key finding concerns the safety of post-osimertinib treatments following the onset of osimertinib-induced ILD; an overall ILD recurrence rate of 16% was associated with the EGFR-TKI rechallenge while a higher rate of 28% was associated with the osimertinib rechallenge. Notably, ILD did not recur in the patients receiving other EGFR-TKIs. The ILD recurrence rate associated with osimertinib therapy was consistent with previous findings, such as those of the Osi-risk Study [8], which reported a 27% recurrence rate. The results of the present study fall between those reported by Li et al. (35%) [6] and Imaji et al. (15%) [3]. The variation in findings across the studies likely stems from differences in the study populations, diagnostic criteria, and follow-up period. Given the higher recurrence rate associated with the osimertinib rechallenge and the relative safety of other TKIs, osimertinib re-administration in patients after the onset of osimertinib-induced ILD requires careful consideration of the benefits and risks on a case-by-case basis. The detailed outcomes, including the efficacy of the non-osimertinib EGFR-TKI rechallenge after first-line osimertinib therapy will be reported in a separate article. It should also be noted that ILD recurrences were often associated with chemotherapy, indicating that caution should be exercised when administering chemotherapy to patients with a history of ILD.

The present study has several, important limitations. First, as an observational study, selection bias cannot be completely ruled out. Patients receiving the EGFR-TKI rechallenge, including those receiving the osimertinib rechallenge, might have had more favorable clinical characteristics which potentially influenced the outcomes. Second, the sample size was relatively small, particularly for the subgroup analyses, thus possibly limiting the generalizability of the findings. Third, the follow-up period might not have been long enough to capture the long-term outcomes and late ILD recurrences fully.

Future research should focus on several, key areas. Larger, prospective studies are needed to validate the findings of the present study and to identify biomarkers capable of predicting ILD development and recurrences. Such markers may help to stratify patients for rechallenges with EGFR-TKIs, including osimertinib, and guide personalized treatment strategies. Additionally, investigating the molecular mechanisms underlying osimertinib-induced ILD may lead to the development of preventive strategies or targeted therapies to mitigate this adverse effect.

## Conclusions

The present study provided important insights into the management of EGFR-mutated NSCLC patients retreated with osimertinib, who had a significantly higher risk of ILD recurrence than those receiving other types of EGFR-TKI. Chemotherapy was also found capable of inducing an ILD recurrence. Therefore, when considering treatment for patients after the onset of osimertinib-induced ILD, the risks and benefits of a rechallenge should be fully weighed, and clinicians should exercise extreme caution while closely monitoring for a possible ILD recurrence.

## Supplementary Material

Supplementary_Caption_hyae178
