# Supplementary material for: Clinical outcomes of patients with EGFR-mutated NSCLC developing interstitial lung disease during first-line osimertinib therapy: a sub-analysis of the Reiwa study
Source: Jpn J Clin Oncol. 2024 Dec 20;55(3):275–82. doi: 10.1093/jjco/hyae178 (PMC11882494; doi:10.1093/jjco/hyae178)
Supplement: Supplementary_Caption_hyae178 [file supplementary_caption_hyae178.docx]

Supplementary Figure 1: Time from the initiation of osimertinib treatment to subsequent treatments.

Supplementary Figure 2: (A) Cumulative incidence of ILD recurrences in all the patients receiving post-osimertinib treatment (n = 59). (B) Cumulative incidence of ILD recurrences in patients receiving the osimertinib rechallenge (n = 18), rechallenge with other EGFR TKIs (n = 13), and non-EGFR TKI rechallenge (n = 28). (C) Cumulative incidence of ILD recurrences in all the patients receiving post-osimertinib treatment stratified by ILD grade.

**Supplementary Table 1.** Second-line therapy in patients with ILD after first-line osimertinib therapy

| **Second-line therapy** | **Patients**  **(N = 59) (%)** |
| --- | --- |
| Osimertinib | 18 (30.4) |
| Carboplatin + pemetrexed | 9 (15.2) |
| Gefitinib | 8 (13.6) |
| Carboplatin + pemetrexed + bevacizumab | 5 (8.5) |
| Afatinib | 3 (5.1) |
| Cisplatin + pemetrexed + bevacizumab | 3 (5.1) |
| Erlotinib | 2 (3.4) |
| Pemetrexed | 2 (3.4) |
| S-1 | 2 (3.4) |
| Carboplatin + nab-paclitaxel | 1 (1.7) |
| Carboplatin + nab-paclitaxel + bevacizumab | 1 (1.7) |
| Carboplatin + S-1 | 1 (1.7) |
| Docetaxel | 1 (1.7) |
| Vinorelbine | 1 (1.7) |
| Atezolizumab | 1 (1.7) |
| Atezolizumab + bevacizumab + paclitaxel + carboplatin | 1 (1.7) |

**Supplementary Table 2.** Characteristics of the 12 patients with ILD relapse after initiation of post-osimertinib treatment

| No. | Age | Sex | Stage | PS  (ECOG) | Smoking  history | Histological type | EGFR mutation | Post-osimertinib treatment  (Second-line treatment) | Initial ILD grade | Relapsed ILD grade | Time from initiation of post-osimertinib treatment to ILD relapse (months) |
| --- | --- | --- | --- | --- | --- | --- | --- | --- | --- | --- | --- |
| 1 | 70 | M | Recurrence | 0 | Never | Adeno | L858R | Osimertinib rechallenge | 1 | 2 | 4.5 |
| 2 | 85 | F | Recurrence | 0 | Never | Adeno | L858R | Osimertinib rechallenge | 1 | 1 | 18.0 |
| 3 | 79 | F | Recurrence | 0 | Never | Adeno | Ex 19 del | Osimertinib rechallenge | 2 | 2 | 4.6 |
| 4 | 79 | F | IV | 1 | Never | Adeno | L858R | Osimertinib rechallenge | 3 | 3 | 0.17 |
| 5 | 72 | M | IV | 0 | Former | Adeno | L858R | Osimertinib rechallenge | 2 | 2 | 0.97 |
| 6 | 72 | F | Recurrence | 0 | Never | Adeno | L858R | Carboplatin + pemetrexed + bevacizumab | 2 | 2 | 5.1 |
| 7 | 70 | M | IV | 1 | Former | Adeno | Ex 19 del | Carboplatin + pemetrexed + bevacizumab | 2 | 2 | 2.5 |
| 8 | 58 | M | Recurrence | 0 | Former | Adeno | Ex 19 del | Carboplatin + pemetrexed | 2 | 2 | 1.3 |
| 9 | 57 | M | Recurrence | 1 | Former | Adeno | Ex 19 del | Carboplatin + pemetrexed | 2 | 2 | 2.1 |
| 10 | 70 | M | IV | 0 | Former | Adeno | Ex 19 del | Carboplatin + S-1 | 3 | 3 | 4.2 |
| 11 | 82 | M | IV | 1 | Never | Adeno | L858R | S-1 | 2 | 2 | 0.2 |
| 12 | 84 | M | Recurrence | 1 | Former | Adeno | Ex 20 ins | Atezolizumab | 1 | 1 | 0.7 |

**Supplementary Table 2.** (continued)

| No. | Third-line treatment | Fourth-line treatment |
| --- | --- | --- |
| 1 | Cisplatin + Pemetrexed + Bevacizumab | ― |
| 2 | ― | ― |
| 3 | Carboplatin + Pemetrexed | Erlotinib + Ramucirumab |
| 4 | ― | ― |
| 5 | ― | ― |
| 6 | Docetaxel + Ramucirumab | ― |
| 7 | S-1 | ― |
| 8 | ― | ― |
| 9 | Erlotinib + Bevacizumab | ― |
| 10 | Docetaxel + Ramucirumab | Pemetrexed |
| 11 | ― | ― |
| 12 | ― | ― |

Abbreviations: ECOG, Eastern Cooperative Oncology Group; PS, performance status; EGFR, epidermal growth factor receptor; ILD, interstitial lung disease; M, male; F, female

**Supplementary Table 3.** Response to second-line treatment

| **Response No. (%)** | **Type of second-line treatment** | | | | | | |
| --- | --- | --- | --- | --- | --- | --- | --- |
|  | Osimertinib  (n = 18) | EGFR-TKI  other than Osimertinib  (n = 13) | Cytotoxic  (n = 17) | Cytotoxic + VEGFI  (n = 9) | ICI  (n = 1) | ICI + cytotoxic + VEGFI  (n = 1) | All patients  (N = 59) |
| CR | 0 (0) | 0 (0) | 0 (0) | 0 (0) | 0 (0) | 0 (0) | 0 (0) |
| PR | 5 (28) | 3 (23) | 4 (23.5) | 3 (33.3) | 0 (0) | 0 (0) | 15 (25.4) |
| SD | 4 (22) | 6 (47) | 9 (53.0) | 5 (55.6) | 0 (0) | 1 (100) | 25 (42.4) |
| PD | 4 (22) | 2 (15) | 3 (17.6) | 1 (11.1) | 1 (100) | 0 (0) | 11 (18.6) |
| NE | 5 (28) | 2 (15) | 1 (5.9) | 0 (0) | 0 (0) | 0 (0) | 8 (13.6) |
| ORR | 27.8% | 23.1% | 23.5% | 33.3% | 0% | 0% | 25.4% |
| DCR | 50% | 69.2% | 76.5% | 88.9% | 0% | 100% | 67.8% |

Abbreviations: EGFR, epidermal growth factor receptor; TKI, tyrosine kinase inhibitor; VEGFI, vascular endothelial growth factor inhibitors; ICI, immune checkpoint inhibitor; CR, Complete Response; PR, Partial Response; SD, Stable Disease; PD Progressive Disease; NE Not Evaluable; ORR, Overall response rate; DCR, Disease control rate
